# A Comparative Assessment of the Nova Stat Profile Prime Plus® Critical Care Analyzer

**DOI:** 10.7759/cureus.9932

**Published:** 2020-08-21

**Authors:** Ali Jawa, Feroza Motara, Muhammed Moolla, Abdullah E Laher

**Affiliations:** 1 Emergency Medicine, University of the Witwatersrand, Johannesburg, ZAF

**Keywords:** point of care testing, poct, emergency department, analyzers, chemistry, nova stat profile prime plus®, radiometer abl 800 flex®, abbott i-stat chem8+®

## Abstract

Background: Point-of-care testing (POCT) plays an integral role in the management of acutely ill patients presenting to the emergency department (ED). Due to its rapid turnaround time, POCT has been shown to improve ED workflow, reduce unnecessary admissions and lessen the burden on ED staff. The aim of the study was to compare the accuracy, precision and linearity of the Nova Stat Profile Prime Plus^®^ (Nova Biomedical, Waltham, MA, USA) to the Radiometer ABL800 FLEX^®^ (Radiometer South Africa Pty Ltd, Gauteng) and the Abbott i-stat Chem8+^®^ (Abbott, Princeton, NJ, USA) POCT analyzers.

Methods: A convenience sample of 150 discarded whole blood specimens was obtained and analyzed. Paired test measurements were conducted for method comparison. Accuracy was measured by pairing individual results from the Nova Stat Profile Prime Plus^®^ with either the Radiometer ABL800 FLEX^®^ or the Abbot i-stat Chem8+^®^ analyzers by calculating the differences.

Results: The with-in run percentage coefficient of variation (%CV) was below 2.4% for pH, carboxyhemoglobin (COHb), deoxyhemoglobin (HHb), total hemoglobin (tHb), total bilirubin (tBil), sodium (Na), potassium (K), chloride (Cl), ionized calcium (iCa), urea, glucose and lactate, and was below 5.1% for all other analytes. The day-to-day %CV was below 1.6% for pH, COHb, HHb, tHb, tBil, Na, K, Cl, iCa, urea, glucose and lactate, and below 6.10% for all other analytes. The correlation coefficient (r) was 0.351 and ranged from 0.897 to 0.998 for all analytes. The mean bias was minimal for all analytes.

Conclusion: There was a good correlation between the Nova Stat Profile Prime Plus^®^ and the Radiometer ABL800 FLEX^®^/Abbott i-STAT Chem8+^®^ POCT analyzers. The Stat Profile Prime Plus^®^ exhibited good precision both within-run and day-to-day.

## Introduction

Technological advances in point-of-care testing (POCT) have revolutionized the practice of emergency medicine over recent years [1,2]. By facilitating rapid clinical decision making and earlier targeted management of patients in the emergency department (ED), POCT has been shown to improve patient flow, reduce length of ED stay, decrease overall staff workload, improve patient satisfaction and reduce costs [3-5].

There has been a significant increase in the production and manufacture of POCT diagnostic devices over the last decade [6]. Prior to the implementation of new devices in the clinical environment, due consideration must be given to several factors including device validity, reliability, performance, durability, practicality, portability, ease of use, sample processing time and cost [7]. Clinical Laboratory Improvement Amendments (CLIA) require a new clinical instrument to undergo rigorous analytical method validation with respect to various performance characteristics that include accuracy, precision, linearity, range, robustness, analytical sensitivity and specificity, detection limits, quantification limits and reference intervals [8].

The Nova Stat Profile Prime Plus® (Nova Biomedical, Waltham, MA, USA) is marketed as a compact maintenance-free POCT biochemical analyzer that is capable of measuring a comprehensive range of biochemical analytes including blood gas parameters, electrolytes, metabolites and co-oximetry from a single sample of blood in less than two minutes [9,10]. In Africa and other resource-limited settings, the implementation of this or similar POCT devices may positively influence patient outcomes by facilitating early appropriate risk stratification and management decisions. In this study, the objective was to evaluate the accuracy of this device by performing a method comparison with specific analytes measured by either the Radiometer ABL800 FLEX® (Radiometer South Africa Pty Ltd, Gauteng) or the Abbott i-STAT Chem8+® (Abbott, Princeton, NJ, USA) analyzer. We also evaluated the precision of the Nova Stat Profile Prime Plus® device by investigating the within-run and day-to-day precision for various analytes.

## Materials and methods

This was a prospective, observational, cross-sectional, comparative validation study conducted at the ED of the Charlotte Maxeke Johannesburg Academic Hospital (CMJAH). CMJAH is a teaching hospital affiliated with the University of the Witwatersrand. Permission to conduct the study was obtained from the hospital manager and the clinical head of the ED. Ethics approval was granted by the Human Research Ethic Committee (Medical) of University of the Witwatersrand (certificate no. M180643). Data was collected on consecutive days between 9 am and 5 pm over a two-week period (May 12-25, 2019).

Table 1 describes the characteristics of the Nova Stat Profile Prime Plus®, Radiometer ABL800 FLEX® and Abbott i-STAT Chem8+® analyzers [9-12]. Sixteen of the 21 analytes measured (pH, partial pressure of carbon dioxide [PCO₂], partial pressure of oxygen [PO₂], oxygen saturation [SO₂], lactate, oxyhemoglobin [O₂Hb], carboxyhemoglobin [COHb], methemoglobin [MetHb], deoxyhemoglobin [HHb], total hemoglobin [tHb], hematocrit [Hct], sodium [Na], potassium [K], chloride [Cl], ionized calcium [iCa] and glucose) were compared with those measured by the Radiometer ABL800 FLEX® analyzer, while urea and creatinine were compared with those measured by the Abbott i-STAT Chem8+® analyzer.

**Table 1:** Description of the three analyzers compared in the study PCO₂, partial pressure of carbon dioxide; PO₂, partial pressure of oxygen; SO₂, oxygen saturation; O₂Hb, oxyhemoglobin; COHb, carboxyhemoglobin; MetHb, methemoglobin; HHb, deoxyhemoglobin; HbF, fetal hemoglobin; tHb, total hemoglobin; Hct, hematocrit; Na, sodium; K, potassium; Cl, chloride; iCa, ionized calcium; iMg, ionized magnesium; Cr, creatinine; Glu, glucose; tBil, total bilirubin; TCO₂, total carbon dioxide

Analyzer [9-12]	FDA approval	Measured analytes	Sample type	Time to sample analysis	Minimum volume of sample required
Nova Stat Profile Prime Plus^®^	2018	21 measured analytes: pH, PCO₂, PO₂, SO₂, lactate, O₂Hb, COHb, MetHb, HHb, HbF, Hct, tHb, Na, K, Cl, iCa, iMg, urea, Cr, Glu, tBil	Heparinized whole blood	90 seconds	135 µL
Radiometer ABL800 FLEX^®^	2004	18 measured analytes: pH, PCO₂, PO₂, SO₂, lactate, O₂Hb, COHb, MetHb, HHb, HbF, tHb, Na, K, Cl, iCa^₂+^, Glu, Cr, tBil	Heparinized whole blood	80 seconds	195 µL
Abbott i-STAT Chem8+^®^	2007	9 measured analytes: Na, K, Cl, TCO₂, iCa, urea, Cr, Glu, Hct	Whole blood	2 minutes	95 µL

Prior to the initiation of data collection, staff members employed at the CMJAH ED were informed of the study aims, objectives and methodology. Consent was obtained from patients or their next of kin if the patient was unable to give consent. Daily device performance checks of all three analyzers were conducted as per manufacturer recommendations. In addition, quality control (QC) checks were also conducted twice daily on each of the study devices. All QC measurements were within the ±2 SD range.

A convenience sample of 150 discarded heparinized arterial or venous blood samples of adult patients (>18 years) who had undergone phlebotomy for routine POCT as part of their clinical management in the ED were analyzed. Samples were thoroughly mixed, and any air bubbles were removed prior to and between instrument comparisons. Paired test measurements of samples were conducted for method comparison. Accuracy was measured by pairing the individual results from the Stat Profile Prime Plus® with either the Radiometer ABL800 FLEX® or the Abbot i-stat Chem8+® and calculating the differences. All the results were recorded on the study data collection sheet.

Precision, which evaluates the ability of the device to produce consistent results over a period of time, can be evaluated by conducting within-run and between-run (day-to-day) measurements on QC material. As per the Clinical Laboratory Standards Institute (CLSI) recommendations, imprecision testing should be conducted at a minimum of two levels of QC material by running at least three replicates over five days [13,14]. For this study, within-run imprecision (expressed as percentage coefficient of variation [%CV]) was determined by assaying five replicates of commercial QC material at different levels whereas day-to-day imprecision (expressed as %CV) was determined by assaying commercial QC material at different levels over five consecutive days. The %CV value was calculated by dividing the mean from the standard deviation of replicate measurements and expressing it as a percentage. The correlation (r) between the Nova Stat Profile Prime Plus® and the corresponding reference analyzer measurements was determined and tabulated.

## Results

A total of 150 discarded blood samples were analyzed. Within-run precision results for all analytes measured with the Nova Stat Profile Prime Plus® analyzer are summarized in Table 2. Within-run %CVs were below 2.4% for pH, COHb, HHB, tHb, tBil, Na, K, Cl, iCa, urea, glucose and lactate and were below 5.1% for all other analytes.

**Table 2:** Within-run imprecision data of the Nova Stat Profile Prime Plus® analyzer %CV, percentage coefficient of variation; PCO₂, partial pressure of carbon dioxide; PO₂, partial pressure of oxygen; SO₂, oxygen saturation; O₂Hb, oxyhemoglobin; COHb, carboxyhemoglobin; MetHb, methemoglobin; HHb, deoxyhemoglobin; tHb, total hemoglobin; tBil, total bilirubin; Na, sodium; K, potassium; Cl, chloride; iCa, ionized calcium; iMg, ionized magnesium

Analytes	Level	n	Mean	SD	%CV
pH	1	10	7.22	0.00	0.03
	2	10	7.41	0.00	0.02
	3	10	7.61	0.00	0.03
PCO₂ (mmHg)	1	10	61.6	2.10	3.41
	2	10	47.0	1.61	3.54
	3	10	22.4	0.40	1.72
PO₂ (%)	1	10	59.5	1.95	3.29
	2	10	99.6	2.14	2.15
	3	10	139.5	6.68	4.79
SO₂ (%)	1	10	60.0	0.00	0.00
	2	10	86.8	2.90	3.34
	3	10	95.0	0.00	0.00
O₂Hb (%)	1	10	28.2	1.44	5.10
	2	10	57.7	0.08	0.15
	3	10	86.7	0.05	0.06
COHb (%)	1	10	24.1	0.07	0.28
	2	10	15.5	0.09	0.61
	3	10	3.7	0.03	0.85
HHb (%)	1	10	18.8	0.07	0.36
	2	10	9.7	0.06	0.59
	3	10	4.8	0.00	0.00
tHb (g/dL)	1	10	20.1	0.07	0.33
	2	10	14.7	0.15	1.06
	3	10	8.6	0.15	0.78
tBil (mg/dL)	1	10	18.7	0.05	0.25
	2	10	8.9	0.03	0.35
	3	10	3.8	0.04	1.12
Na (mmol/L)	4	10	142.4	0.42	0.32
	5	10	120.2	1.54	1.42
K (mmol/L)	4	10	3.9	0.01	0.23
	5	10	6.1	0.09	1.41
Cl (mmol/L)	4	10	126.6	0.70	0.53
	5	10	100.1	0.83	0.84
iCa (mmol/L)	4	10	1.1	0.00	0.39
	5	10	1.5	0.03	2.32
iMg (mmol/L)	4	10	0.6	0.00	3.92
	5	10	1.2	0.10	5.01
Urea (mmol/L)	4	10	6.1	0.00	0.72
	5	10	19.3	0.32	1.53
Creatinine (mmol/L)	4	10	82.6	3.91	4.82
	5	10	583.0	7.73	1.35
Glucose (mmol/L)	4	10	5.4	0.00	0.96
	5	10	16.3	0.03	1.87
Lactate (mmol/L)	4	10	2.0	0.00	0.00
	5	10	6.9	0.12	0.81

Day-to-day precision results for all analytes measured with the Nova Stat Profile Prime Plus® analyzer are summarized in Table 3. The %CV was below 1.6% for pH, COHb, HHB, tHb, tBil, Na, K, Cl, iCa, urea, glucose and lactate and below 6.10% for all other analytes.

**Table 3:** Day-to-day imprecision data of the Nova Stat Profile Prime Plus® analyzer %CV, percentage coefficient of variation; PCO₂, partial pressure of carbon dioxide; PO₂, partial pressure of oxygen; SO₂, oxygen saturation; O₂Hb, oxyhemoglobin; COHb, carboxyhemoglobin; MetHb, methemoglobin; HHb, deoxyhemoglobin; tHb, total hemoglobin; tBil, total bilirubin; Na, sodium; K, potassium; Cl, chloride; iCa, ionized calcium; iMg, ionized magnesium

Analytes	Level	n	Mean	SD	%CV
pH	1	10	7.20	0.002	0.01
	2	10	7.39	0.002	0.02
	3	10	7.62	0.002	0.00
PCO₂ (mmHg)	1	10	57.8	3.35	5.83
	2	10	40.9	2.25	5.43
	3	10	19.7	0.80	4.12
PO₂ (%)	1	10	54.9	1.73	2.96
	2	10	91.2	2.59	2.77
	3	10	128.0	4.82	3.61
SO₂ (%)	1	10	62.1	0.01	0.03
	2	10	84.6	3.80	3.56
	3	10	97.2	0.03	0.05
O₂Hb (%)	1	10	26.3	1.80	4.09
	2	10	61.7	0.12	0.25
	3	10	83.9	0.03	0.09
COHb (%)	1	10	26.4	0.06	0.32
	2	10	14.4	0.09	0.67
	3	10	3.9	0.04	0.82
HHb (%)	1	10	20.1	0.07	0.38
	2	10	9.4	0.08	0.61
	3	10	4.0	0.01	0.01
tHb (g/dL)	1	10	19.7	0.09	0.48
	2	10	24.3	0.18	0.90
	3	10	8.9	0.06	0.81
tBil (mg/dL)	1	10	19.9	0.06	0.28
	2	10	8.5	0.03	0.39
	3	10	4.2	0.05	1.18
Na (mmol/L)	4	10	141.2	0.16	0.11
	5	10	116.3	0.19	0.21
K (mmol/L)	4	10	3.9	0.004	0.13
	5	10	6.2	0.008	0.17
Cl (mmol/L)	4	10	128.2	1.47	1.18
	5	10	99.0	0.63	0.65
iCa (mmol/L)	4	10	1.1	0.004	0.42
	5	10	1.5	0.005	0.04
iMg (mmol/L)	4	10	0.6	0.009	1.42
	5	10	1.2	0.02	2.03
Urea (mmol/L)		10	7.3	0.13	0.92
	5	10	23.1	0.14	1.84
Creatinine (mmol/L)		10	84.3	3.34	3.97
	5	10	583.6	13.81	2.43
Glucose (mmol/L)	4	10	4.5	0.06	0.05
	5	10	15.8	0.34	1.62
Lactate (mmol/L)		10	2.0	0.00	0.06
	5	10	6.9	0.12	1.62

Table 4 describes the correlation between the Nova Stat Profile Prime Plus® analyzer and the Radiometer ABL800 FLEX®/Abbott i-STAT Chem8+® analyzers. The correlation coefficient ranged from 0.898 to 0.998 for all analytes.

**Table 4:** Correlation data for the Nova Stat Profile Prime Plus® analyzer PCO₂, partial pressure of carbon dioxide; PO₂, partial pressure of oxygen; SO₂, oxygen saturation; O₂Hb, oxyhemoglobin; COHb, carboxyhemoglobin; MetHb, methemoglobin; HHb, deoxyhemoglobin; tHb, total hemoglobin; tBil, total bilirubin; Na, sodium; K, potassium; Cl, chloride; iCa, ionized calcium

Analyte	Analyzer	Minimum	Maximum	Correlation (r)
pH	Radiometer ABL800 FLEX^®^	6.74	7.60	0.992
	Nova Stat Profile Prime Plus^®^	6.71	7.59	
PCO₂	Radiometer ABL800 FLEX^®^	17.00	71.90	0.945
	Nova Stat Profile Prime Plus^®^	14.80	83.70	
PO₂	Radiometer ABL800 FLEX^®^	13.60	178.00	0.994
	Nova Stat Profile Prime Plus^®^	18.30	180.20	
SO₂	Radiometer ABL800 FLEX^®^	34.20	99.70	0.994
	Nova Stat Profile Prime Plus^®^	30.00	99.00	
Lactate	Radiometer ABL800 FLEX^®^	0.40	17.00	0.998
	Nova Stat Profile Prime Plus^®^	0.40	18.30	
O₂Hb	Radiometer ABL800 FLEX^®^	12.70	98.80	0.998
	Nova Stat Profile Prime Plus^®^	7.20	97.70	
COHb	Radiometer ABL800 FLEX^®^	0.20	11.30	0.909
	Nova Stat Profile Prime Plus^®^	0.30	10.00	
HHb	Radiometer ABL800 FLEX^®^	0.30	33.90	0.994
	Nova Stat Profile Prime Plus^®^	1.00	39.70	
tHb	Radiometer ABL800 FLEX^®^	4.70	19.70	0.990
	Nova Stat Profile Prime Plus^®^	5.30	19.90	
Hematocrit	Radiometer ABL800 FLEX^®^	15.00	60.20	0.935
	Nova Stat Profile Prime Plus^®^	16.00	58.00	
Na	Radiometer ABL800 FLEX^®^	111.00	165.00	0.898
	Nova Stat Profile Prime Plus^®^	115.50	164.00	
K	Radiometer ABL800 FLEX^®^	2.20	6.30	0.987
	Nova Stat Profile Prime Plus^®^	2.40	6.48	
Cl	Radiometer ABL800 FLEX^®^	80.00	124.00	0.953
	Nova Stat Profile Prime Plus^®^	87.40	120.90	
iCa	Radiometer ABL800 FLEX^®^	0.57	1.99	0.969
	Nova Stat Profile Prime Plus^®^	0.71	2.00	
Glucose	Radiometer ABL800 FLEX^®^	3.10	21.00	0.992
	Nova Stat Profile Prime Plus®	3.50	22.40	
Urea	Abbott i-STAT Chem8+^®^	1.00	29.80	0.982
	Nova Stat Profile Prime Plus®	1.70	30.70	
Creatinine	Abbott i-STAT Chem8+^®^	19.0	1030.00	0.972
	Nova Stat Profile Prime Plus®	51.0	758.00	

## Discussion

Everyday many patients present to the ED in a critical state requiring quick decision making [5], with POCT playing a crucial role in the management of these patients. POCT testing is laboratory diagnostic testing performed at or near the site where clinical care is delivered. The key objective of POCT is to acquire rapid and accurate results thereby facilitating timeous and appropriate management [15].

Specimens sent to the central laboratory are generally subjected to prolonged delays in acquiring results. Additionally, potential alteration may occur to samples due to the instability of the analyte during transport, environmental exposure, cellular metabolism and temperature variation [16]. This may result in inaccurate findings and can potentially impact patient care. Since POCT is initiated and performed rapidly once the sample is collected, the potential of sample deterioration is reduced.

POCT is a rapidly evolving field with an annual growth rate of 7%-8%. In Africa, less than 500 laboratories fulfill the criteria for international standard requirements, while less than one laboratory professional is available for every 10,000 people [17]. A single, reliable and cost-effective analyzer providing timely and reliable results of an expanded panel of analytes will be of value not only in low- and middle-income countries, but in any ED setting too.

Recent advances in medical technology have greatly expanded the capacity of POCT systems. Modern POCT analyzers are capable of measuring an extended panel of biochemical analytes in a short period of time and wirelessly transmitting results to synchronize with electronic patient medical records [12].

The Nova Stat Profile Prime® is a POCT analyzer that has been in use since 2014 and is capable of measuring and calculating 10 biochemical parameters in 60 seconds. In comparison, the Nova Stat Profile Prime Plus® was introduced in 2017, offering an expanded profile of 22 parameters with a shorter duration to complete analysis of the sample. The Nova Stat Profile Prime Plus® has individual cartridges in comparison to other POCT devices, offering a significant advantage in analyzer uptime compared to combined sensor/calibrator systems [9].

In this study, the Nova Stat Profile Prime Plus® POCT analyzer was compared with other established validated analyzers in current use at the study site. We compared various blood gas parameters, electrolytes, metabolites and co-oximetry variables, most of which are essential for the management of critically ill patients in an ED setting.

Our study showed that agreement targets for all analytes were met when compared to the method of comparison. The correlation coefficient of all analytes measured ranged from 0.898 to 0.998. As per the Westgard classification, a correlation coefficient ranging from 0.90 to 1.00 can be regarded as very high, while a correlation coefficient ranging from 0.70 to 0.89 can be regarded as high [18]. Additionally, the analyte concentration directly impacts on the correlation coefficient, with a wider analyte range having a greater r value [19]. Therefore, in this study, values of r for all analytes were suggestive of a good correlation. Furthermore, the %CVs for all the analytes tested were acceptable, indicating that the imprecision of the Nova Stat Profile Prime Plus® was minimal and in accordance with the manufacturer's specifications.

This study had few limitations. Firstly, it was not possible to obtain extremely high and low ranges for all the analytes. Secondly, although every attempt was made to process samples with minimal time delays, there is a possibility that air exposure due to slight time delays could have altered blood gas levels [20]. Thirdly, limitations associated with convenience sampling methodology such as selection bias and researcher bias may also be applicable to this study.

## Conclusions

The Nova Stat Profile Prime Plus® correlated well with the Radiometer ABL800 FLEX® and the Abbott i-STAT Chem8+® in both high and low ranges. The Stat Profile Prime Plus® exhibited good precision both within-run as well as day-to-day. Hence, it can be concluded that the Nova Stat Profile Prime Plus® is a reliable device that is capable of analyzing a comprehensive panel of analytes in the ED setting.
